# Role of Endogenous Regulators of Hem- And Lymphangiogenesis in Corneal Transplantation

**DOI:** 10.3390/jcm9020479

**Published:** 2020-02-09

**Authors:** Thomas Clahsen, Christian Büttner, Niloofar Hatami, André Reis, Claus Cursiefen

**Affiliations:** 1 Department of Ophthalmology, University of Cologne, Faculty of Medicine and University Hospital Cologne, 50937 Cologne, Germany; niloofar.hatami@uk-koeln.de (N.H.); claus.cursiefen@uk-koeln.de (C.C.); 2 Center for Molecular Medicine (CMMC), University of Cologne, 50937 Cologne, Germany; 3 Institute of Human Genetics, University Hospital Erlangen, Friedrich-Alexander-Universität Erlangen-Nürnberg, 91054 Erlangen, Germany; Christian.Buettner@uk-erlangen.de (C.B.); andre.reis@uk-erlangen.de (A.R.)

**Keywords:** cornea transplantation, graft rejection, lymphangiogenesis, modulators of lymphangiogenesis

## Abstract

Under normal conditions, the cornea, being the transparent “windscreen” of the eye, is free of both blood and lymphatic vessels. However, various diseases of the eye, like infections, can interfere with the balance between promoting and inhibiting factors, which leads to ingrowth of blood and lymphatic vessels. The newly formed lymphatic vessels increase the risk of graft rejection after subsequent corneal transplantation. Corneal transplantation is one of the most commonly performed transplantations worldwide, with more than 40,000 surgeries per year in Europe. To date, various anti-hem- and anti-lymphangiogenic treatment strategies have been developed specifically for the corneal vascular endothelial growth factor (VEGF) pathway. Currently, however, no treatment strategies are clinically available to specifically modulate lymphangiogenesis. In this review, we will give an overview about endogenous regulators of hem- and lymphangiogenesis and discuss potential new strategies for targeting pathological lymphangiogenesis. Furthermore, we will review recently identified modulators and demonstrate that the cornea is a suitable model for the identification of novel endogenous modulators of lymphangiogenesis. The identification of novel modulators of lymphangiogenesis and a better understanding of the signaling pathways involved will contribute to the development of new therapeutic targets for the treatment of pathological lymphangiogenesis. This, in turn, will improve graft rejection, not only for the cornea.

## 1. Introduction

**Figure 1:**
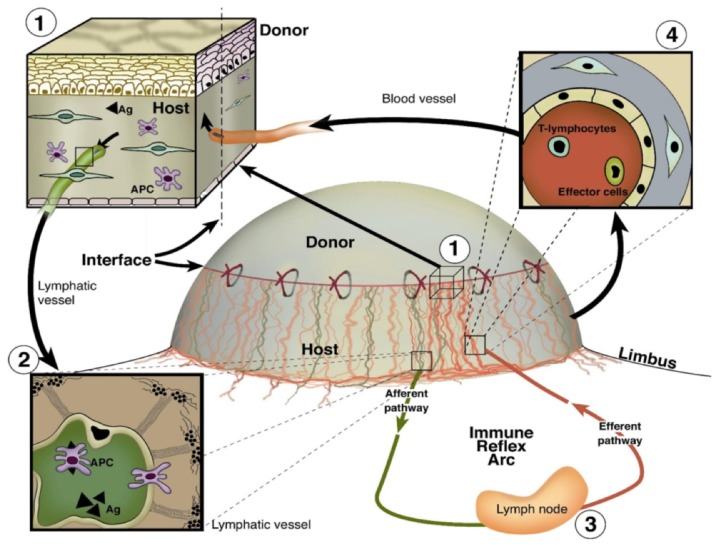
Important role of blood and lymphatic vessels in the high-risk corneal host bed as exit and entry routes of the immune reflex arc leading to immunologic graft rejection. (1) Magnification of the host–graft interface where blood (red) and lymphatic (green) vessels reach the graft. Antigen (Ag) and antigen-presenting cells (APCs) both of host and donor can leave the cornea using corneal lymphatics. (2) and migrate through corneal lymphatic vessels to the draining lymph nodes. (3) After stimulation of immune effector cells in the regional lymph node, T lymphocytes/effector cells can be released via the efferent blood vessels (4) and gain direct access to the transplant and initiate a rejection reaction (efferent arm of the immune reflex arc). (adopted from [4]).

The cornea is a well-established model to analyze the mechanism underlying (lymph)angiogenesis. In its normal condition, the cornea is devoid of blood and lymphatic vessels [1]. The blood and lymph vessels coming from the conjunctiva terminate in the limbal region, the border between the vascularized conjunctiva and avascular cornea. A further advantage of the cornea is its transparency and exposed position, enabling easy visualization and imaging of pathological corneal neovascularization. The precise balance of pro- and anti-(lymph)angiogenic factors play a very important role in maintaining this avascularity and transparency. A number of pathologic insults, such as inflammation, infection, trauma, and chemical burns [2,3] lead to an imbalance between pro- and anti-(lymph)angiogenic factors. This imbalance can cause the ingrowth of blood and lymphatic vessels into the cornea which not only results in reduced vision but also increases the risk for immune reactions after necessary cornea transplantation. The corneal immune privilege is important for the success of corneal transplantation and closely relates to the avascular nature of the cornea. The grafts, transplanted into an avascular corneal recipient bed (normal-risk keratoplasty), show a five-year survival rate of over 90%. In contrast, grafts, implanted into pathologically prevascularized murine corneal recipient beds (high-risk keratoplasty) (Figure 1), are rejected in more than 50% of transplantations despite immunosuppressive therapy [4,5]. Therefore, treatments prior to transplantation that reduce the blood and lymphatic vessel load in inflammation would be a great benefit.

In this review, we briefly discuss the known endogenous regulators of lymphangiogenesis and present the currently available methods for inhibiting the growth of blood and lymphatic vessels or inducing their regression, which are already partially clinically used. Subsequently, we will review the newly identified regulators of lymphangiogenesis and finally, we want to highlight that the cornea is an ideal model for identifying new modulators of lymphangiogenesis.

## 2. Endogenous Regulators of (lymph)angiogenesis

Various molecular mechanism contributes to the corneal (lymph)angiogenic privilege. Up to now, various endogenous factors that promote or inhibit lymphangiogenesis have been identified. Angiogenesis and lymphangiogenesis are mainly induced by members of the vascular endothelial growth factor (VEGF) family. Binding of VEGF-A and VEGF-B to VEGFR-1 and VEGFR-2 induces hemangiogenesis [6,7,8]. In addition, the cytokines platelet-derived growth factor (PDGF) [9], fibroblast growth factor-2 (FGF-2), placental growth factor (PIGF), hepatocyte growth factor (HGF) [10] and the adapter protein insulin receptor substrate-1 (IRS-1) [11] have also been found to induce hemangiogenesis.

Lymphangiogenesis is mainly induced by binding of VEGF-C and VEGF-D to VEGFR-2 and VEGFR-3 [6,7,12]. However, the pro-angiogenic factor VEGF-A has also been shown to be capable of inducing lymphangiogenesis. This effect can be mediated either indirectly by the recruitment of VEGFR-1 positive macrophages, which then secrete VEGF-C and D, or directly by proliferative action on the lymphatic endothelial cells [13,14,15]. As with angiogenesis, there are also other growth factors, such as FGF-2 [16], PDGF [17], angiopoietin [18,19] and HGF [20], that induce lymphangiogenesis. Moreover, in a wide range of diseases, such as cancer, cardiovascular disease, and inflammation, pro-inflammatory or anti-inflammatory cytokines may induce or suppress lymphangiogenesis [21,22]. Pro-inflammatory cytokines, like interleukin (IL)-1, IL-12, IL-18, tumor necrosis factor (TNF)-α, and interferon (IFN)-β, are produced mainly by macrophages but also by NK cells, T cells, B cells and neutrophils [23,24]. Cytokines such as IL-1 and TNF have been shown to induce VEGF-C expression and thus promote lymphangiogenesis [25]. On the other hand, anti-inflammatory cytokines like IL-4, IL-10, IL-11 and IL-13 are secreted by macrophages, as well as T cells, vascular smooth muscle cells and endothelial cells [23]. It was also described that the anti-inflammatory cytokines IL-4 and IL-13 downregulate the prospero homeobox 1 (Prox1) transcription factors in lymphatic endothelial cells (LECs), thus impairing lymphatic endothelial cell survival, proliferation, and migration [26,27].

Endogenous inhibitors play an important regulatory role in preserving the angiogenic privilege and inhibiting and regressing blood and lymphatic vessels induced by minor vascular stimuli. These endogenous antiangiogenic factors can be categorized into endostatin/endostatin analogues (endostatin, arresten, and tumstatin), plasminogen/serine protease inhibitors (angiostatin and pigment epithelial-derived factor [PEDF]), thrombospondin-1, -2 and soluble VEGF receptors [28,29,30,31,32].

Major components of the cornea are collagen fibrils of uniform diameter and proteoglycan interacting with the collagen fibers [33,34]. The stromal proteoglycans belong to the group of small leucine-rich proteins (SLPR). The SLRP has key functions in collagen fibril growth and organization, extracellular matrix (ECM) assembly, corneal transparency and in the regulation of inflammatory processes [35]. For lumican, biglycan, and decorin, the main components of the corneal SLRP, it is also discussed that they regulate the macrophage and neutrophil migration and cytokine secretion in the cornea [35,36].

Fragments of the ECM collagens play a functional role in the angiogenesis process. Endostatin consists of a 20-kDa C-terminal fragment of type XVIII collagen of the ECM and is one of the most potent inhibitors of angiogenesis. It binds to different integrins (α_3_β_1_, α_5_β_1_, α_v_β_3_, and α_v_β_5_)_,_ inhibits cell proliferation, and disrupts cell migration [32,37,38,39]. Tumstatin, a 28-kDa fragment of collagen IV, binds to integrin α_v_β_3_ and α_v_β_5_ and contains anti-angiogenic capacity. The binding to integrin α_v_β_3_ is necessary for the antiangiogenic activity [40,41]. Another fragment of type collagen IV with anti-angiogenic properties is arresten. Arresten is the 26-kDa fragment of the C-terminal domain of type IV collagen mediating its anti-angiogenic effects via binding to integrin α_1_β_1_ [41].

Angiostatin, an endogenous antiangiogenic factor that is cleaved from plasminogen produced in the cornea, can attach to several surface proteins in vascular endothelial cells and hinder their migration and tubule formation [42,43].

Thrombospondins (TSPs) belongs to a family of high molecular weight glycoproteins that are secreted by most cell types and participate in cell-to-cell and cell-to-matrix communication. TSP-1 was the first naturally occurring protein inhibitor of angiogenesis to be identified. We have observed that TSP-1 is involved in the inhibition of corneal hemangiogenesis in vivo [44]. In this context, it has been shown that TSP-1 can inhibit hemangiogenesis both directly and indirectly [31,45]. Subsequent studies have shown that also TSP-2 inhibits angiogenesis [31].

Moreover, the cornea also expresses soluble variants of VEGFR-1, -2 and -3 which act as decoy receptors. The soluble VEGFR-1 lacks the transmembrane and tyrosine kinase domains [46] and traps secreted VEGF-A [28].

The corneal epithelium also plays a central role in maintaining the alymphatic state of the cornea. The corneal epithelium expresses the soluble forms of VEGFR-2 and -3 (sVEGFR-2, sVEGFR-3) [47,48]. The sVEGFR-2 is the monomer of the extracellular domain of the membrane-bound receptor. This soluble form results from alternative splicing and acts as an important endogenous antagonist by capturing free monomeric VEGF-C and thus preventing lymphangiogenesis [47]. On the other hand, the sVEGFR-3 is the truncated isoform of the VEGFR-3, consisting only of the extracellular ligand-binding domain of VEGFR-3 [48]. The binding of VEGF-C to sVEGFR-3 not only inhibits lymphangiogenesis [48,49], but also causes a regression of already formed lymphatic vessels [49]. The membrane-bound VEGFR-3 is an endothelium-specific receptor tyrosine kinases expresses in the fetal vasculature in venous and lymphatic endothelium. This is restricted to lymphatic endothelium in adults. However, we could show that membrane-bound VEGFR-3 expressed on the human corneal epithelial cells [1] also binds VEGF-C and thus helps maintain corneal avascularity [50].

Recent and ongoing research demonstrates that immune-mediated corneal graft rejection depends on both angiogenesis and lymphangiogenesis. Several studies show that anti-lymphangiogenic treatment exhibits success in improving graft survival [3,47,51]. Therefore, the identification of novel endogenous regulators contributes to a better understanding of the role of angiogenesis and lymphangiogenesis in corneal transplants, which subsequently has the potential to reduce graft rejection rates.

## 3. Endogenous Regulators of Lymphangiogenesis in Corneal Transplantation

During the past years, research has demonstrated that modulating both VEGFs and their receptors in both low- and high-risk corneal transplantation can reduce angiogenesis and lymphangiogenesis [47,51,52,53]. The potential for neovascularization can thereby be reduced and the chance of transplant survival can be increased. The same potentially also works for other forms of transplantation.

In this context, we were able to show that the use of VEGF-Trap_(R1R2)_ to neutralize VEGF-A early postoperatively significantly reduced both hem- and lymphangiogenesis and significantly promoted long-term graft survival in a normal-risk corneal transplantation model [52]. It has recently shown that the VEGF-Trap significantly inhibits the infiltration of immune cells, including macrophages and CD3^+^ T cells. [54]. The subconjunctival administration of Bevacizumab, a neutralizing antibody for all VEGF-A isoforms, significantly diminished corneal graft opacity, prolonged the graft survival, and reduced the neovascular area and invasion area compared to topical treatment and no treatment (control) in a mouse model of high-risk corneal transplantation [55].

This approach has been transferred to the clinic and several studies have shown promising results following Bevacizumab treatment for corneal neovascularization in patients before and/or after penetrating keratoplasty. So, it could be shown that intrastromal application [56,57,58], topical application [59,60,61] and subconjunctival injection [62] respectively of Bevacizumab leads to a reduction in corneal neovascularization and so enables long term success of subsequent corneal transplantation after high-risk transplantation. Just recently, we demonstrated that the fine-needle diathermy in combination with subconjunctival injection of Bevacizumab prior to high-risk keratoplasty in patients results in graft survival rates comparable to survival rates seen in normal-risk keratoplasty [63]. Moreover, a combined subconjunctival and topical Bevacizumab treatment improves the graft survival rate in 70% of high-risk transplantations during three years of follow-up [60].

Another approach that has been used in clinical trials to suppress the growth of lymphatic vessels is the blockade of the insulin receptor substrate (IRS-1). IRS-1 is a cytosolic adapter protein without intrinsic kinase activity that recruits other proteins to their receptors [64]. Using antisense oligonucleotide against IRS-1 we have observed reduced proliferation of human dermal lymphatic endothelial cells. The additional blockade of IRS-1 reduces the expression of VEGF-A, but not the expression of VEGF-C, VEGF-D, and VEGFR-3. Treatment with IRS-1 antisense oligonucleotide also inhibits inflammation-induced lymphangiogenesis in a murine suture-model [53]. The IRS-1 antisense oligonucleotide has now passed through phase II and III trials and demonstrates a significant inhibition of corneal neovascularization in patients [65].

In addition to the inhibitors of angiogenesis described above, which have meanwhile found use in the clinic, some endogenous inhibitors of lymphangiogenesis are also known. In recent years, however, these have only been studied preclinically for their potential of improving graft survival.

The role of lymphatics in transplantation and the effects of VEGF-C and VEGF-D blockade on soluble VEGFR-3 (sVEGFR-3) on corneal neovascularization and graft survival have recently been investigated. The treatment with sVEGFR-3 resulted in a significant blockade of lymphangiogenesis two weeks after transplantation. Moreover, significantly prolonged corneal allograft survival compared to the control group at eight weeks after transplantation could be observed. This was associated with significantly reduced frequencies of allosensitized T cells and decreased frequencies of IFN-γ-producing CD4^+^ T cells [51]. There is evidence that the primary cellular mediators of immune rejection are predominantly mediated by CD4^+^ T cells [66].

The blockade of VEGF-C by sVEGFR-2 is essential for corneal alymphaticity. Moreover, intracorneal administration of sVEGFR-2 reduced lymphangiogenesis, enhanced corneal allograft survival and suppressed lymphangioma cellular proliferation in a corneal transplantation model [47].

**Figure 2:**
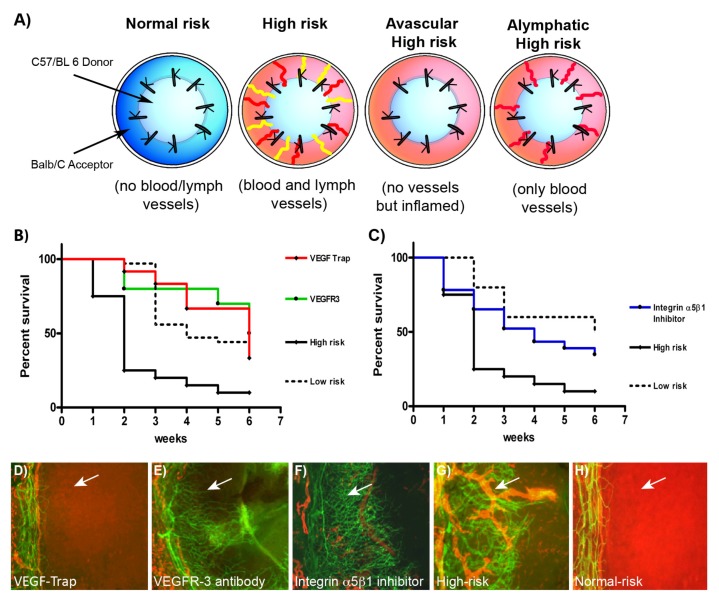
Lymphatic vessels in the recipient bed prior to transplantation determine graft survival. (**A**) Schematic diagram showing transplantation models normal-risk (avascular), high-risk (inflamed and hemvascularized and lymphvascularized), avascular high-risk (inflamed, avascular), and alymphatic high-risk recipient beds (inflamed and hemvascularized, but no lymphatic vessels). (**B**) and (**C**) Kaplan–Meier survival curve. To induce inflammation the suture-induced corneal neovascularization assay was performed two weeks prior transplantation and mice were treated with vascular endothelial growth factor (VEGF)-Trap_R1R2_ (***B*** (red line) and ***D***; no blood and lymphatic vessels, but reduced inflammation in the recipient bed), VEGFR-3 Ab (mF4-31C1) (***B*** (green line) and ***E***; only blood vessels present in the recipient bed), or integrin α_5_β_1_ inhibitor (JSM6427) (***C*** (blue line) and ***F***; only blood vessels present in the recipient bed). Graft survival was compared with prehemvascularized and prelymphvascularized controls (***B*** and ***C*** (black line), ***G***: “high-risk” recipient bed) and avascular recipient controls (***B*** and ***C*** (dotted line), ***H***: “low-risk” recipient bed). The graft survival was significantly better when transplants were placed into recipient beds lacking lymphatic vessels compared with beds with lymphatic vessels present. (VEGF-Trap versus high-risk: *p* < 0.0001; VEGFR-3 versus high-risk: *p* < 0.0002; *n* = 10; JSM6427 versus high-risk: *p* < 0.032, *n* = 23; Kaplan–Meier survival curve). (***D**–**H***) Corneal whole mounts stained for blood vessels (CD31, green) and for lymphatic vessels (LYVE-1, red) in different recipient beds. Representative images of recipient corneal beds at the time of transplantation after corneas were treated with (**D**) VEGF-Trap_R1R2_, (**E**) VEGFR-3 (mF4-31C1), (**F**) integrin α_5_β_1_ inhibitor (JSM6427), or (**G**) untreated high-risk and (**H**) normal-risk (original magnification × 100). Arrow, prevascularized cornea. (modified from [3]).

To investigate the role of existing lymphatic vessels in graft rejection the murine model of corneal transplantation [3] with different experimental conditions in the recipient was used: 1. normal-risk (healthy avascular recipient bed), 2. high-risk (recipient bed vascularized with blood and lymphatic vessels) and 3. alymphatic high-risk (recipient bed vascularized only with blood vessels, the outgrowth of lymphatic vessels was inhibited by the VEGFR-3 Ab mF4-31C1 treatment) (Figure 2A and 2B). Target inhibition of lymphangiogenesis during the inflammatory vascularization phase prior to corneal transplantation the survival of the graft used in the alymphatic bed was significantly improved compared to the graft used in the high-risk recipient bed. Even more, the survival of the graft under alymphatic conditions was comparable to the survival under low-risk conditions [3].

Also blocking VEGF-C with an anti-VEGF-C (VGX-100) antibody in a murine model of high-risk transplantation showed effectively reduced graft lymphangiogenesis in comparison to the untreated control group. This treatment significantly improved the eight-week graft survival compared to control [54]. Furthermore, the administration of anti-VEGF-C not only suppresses the corneal angiogenic responses but also inhibits trafficking and maturation of APCs [67].

Podoplanin, a mucine-type glycoprotein, is highly expressed on lymphatic endothelial cells but not on arterial and venous endothelial cells in the vascular system. The surface receptor C-type lectin-like receptor 2 (CLEC-2) is expressed on dendritic cells, neutrophils, and platelets and binds to podoplanin [68,69]. Moreover, podoplanin also binds CCL21 with high affinity [70] and this interaction has implications for lymphocyte trafficking, as LECs express CCL21 to direct lymphocyte and DC trafficking to the LNs [71]. Recently, the influence of podoplanin on graft rejection has been examined. The administration of anti-podoplanin antibodies both inhibits the growth of the lymphatic vessels and drastically reduces the infiltration of macrophages. Moreover, the rejection reaction in the corneal transplantation model is also significantly suppressed [72].

Integrins are a family of heterodimeric transmembrane glycoproteins mediating cell-cell and cell-ECM connections. Some of the integrins play an important role in angiogenesis or lymphangiogenesis. However, the integrins that regulate lymphangiogenesis are different from those that regulate angiogenesis [73]. Integrin α_9_β_1_ plays a critical role in lymphangiogenesis, since integrin α_9_β_1_ promotes VEGF-C and D stimulated cell migration by directly binding these growth factors [8,74]. In order to analyze the function of integrin α_9_β_1_, corneal transplantation between fully mismatched mice was performed. The recipients received subconjunctival injections of either integrin α_9_ blocking antibody or isotype control twice a week for eight weeks. The treatment with an anti-integrin α_9_ blocking antibody does not affect corneal lymphangiogenesis. However, they demonstrated that the treatment suppressed lymphatic valvulogenesis after transplantation whereby a higher rate of graft survival was achieved [75].

Integrin α_5_β_1_ is expressed by a subpopulation of lymphatic vessels in the inflamed cornea [3], but it does not appear to play a role in tumor lymphangiogenesis [76]. To analyze the impact of the integrin α_5_β_1_ in high-risk corneal transplantation, the small-molecule antagonists JSM6427 was used to inhibit integrin α_5_β_1_ function. The treatment with the antagonists led to specific inhibition of lymphangiogenesis, while hemangiogenesis was not affected significantly. Thus, the systemic inhibition of integrin α_5_β_1_ and, thereby, the absence of lymphatic vessels in the recipient bed significantly improved corneal graft survival after high-risk corneal transplantation compared to control and reduced the risk for rejection to the level of low-risk corneal transplantation [3] (Figure 2C).

Semaphorins, initially described as axon guidance factors, have recently been implicated in a variety of physiological and developmental functions including regulation of immune response, angiogenesis, and migration of neural crest cells [77]. Members of the class 3 semaphorins are soluble ligands that bind to the neuropilin and plexin receptors [78,79]. Semaphorin-3F (Sema-3F) a member of the class 3 semaphorins is a known mediator contributing to the anti-angiogenic barrier in the eye [80,81]. Recently, a strong expression of *Sema3F* in the corneal epithelium of both naive murine and healthy human cornea could be detected. However, under inflamed condition, Sema-3F was significantly downregulated. Topical application of recombinant Sema-3F significantly inhibits the outgrowth of corneal lymphatic vessels and increases the graft survival in the murine model of high-risk corneal transplantation [82].

In conclusion, the blockade of podoplanin, the inhibition of integrin or the treatment with Sema-3F could be used as promising new therapeutic targets in improving graft rejection.

## 4. Identification of Novel Endogenous Regulators of Lymphangiogenesis

### 4.1. Proteins and Peptides in Lymphangiogenesis

In recent years, only a few novel endogenous modulators of lymphangiogenesis have been identified. Some of these were already known inhibitors of angiogenesis, in which now an inhibitory function in lymphangiogenesis was also determined. Additionally, we and others were able to further identify new regulators of lymphangiogenesis. These regulators help to better understand the regulation of lymphangiogenesis. In the cornea, beside the above mentioned sVEGFR-2 [47], sVEGFR-3 (sVEGFR-3) [48,49], and the membrane-bound VEGFR-3, thrombospondin (TSP)-1 [83], vasohibin-1 [84] and neuropilin (NP-2) [85] were also identified and accepted as endogenous inhibitors.

We were able to show that TSP-1 inhibits not only hemangiogenesis but also lymphangiogenesis. TSP-1 binds to CD36 on macrophages and leads to an inhibition of VEGF-C production in these macrophages, which in turn leads to an inhibition of lymphangiogenesis [83].

Vasohibin-1 (VASH1), a novel inhibitor of angiogenesis is selectively expressed in endothelial cells (EC). Its expression is induced by growth factors such as VEGF and FGF-2 and it inhibits the migration, proliferation, and tube formation of ECs [86]. Recently, it was observed that vasohibin-1 also inhibited VEGF-C-stimulated lymphangiogenesis supports a direct anti-lymphangiogenesis activity of vasohibin-1 [84].

Neuropilin-2 (NP-2) is associated with VEGFR-3 and mediates lymphatic vessel sprouting in response to VEGF-C [85]. The artificial microRNA (amiRNA) targeting NP-2 has been shown to efficiently reduced NP-2 expression in lymphatic endothelial cells. Furthermore, the subconjunctival application of NP-2 amiRNA improved graft survival in high-risk transplantation model [87].

Matrix metalloproteinases (MMPs) are endopeptidases essential for tissue remodeling and signal transduction in processes ranging from growth and development to cancer progression, metastasis, and angiogenesis [88,89]. Membrane type-1-matrix metalloproteinase (MT1-MMP) is a membrane-bound metalloproteinase that is essential for diverse physiological processes like extracellular matrix remodeling and pericellular proteolysis [90]. The cleavage of VEGFR-1 by corneal MT1-MMP results in a VEGF-Trap effect that reduces the proangiogenic effect of VEGF-A_165_ and thus corneal angiogenesis [91]. Furthermore, MT1-MMP deficient mice have defective fibroblast growth factor-2 (FGF2) induced corneal angiogenesis [92,93]. So, MT1-MMP has been identified as a crucial regulator of blood vessel growth. It has been recently shown that MT1-MMP directly cleaves LYVE-1 on lymphatic endothelial cells and thereby inhibits LYVE-1-mediated lymphangiogenic responses. Therefore, MT1-MMP is also an endogenous inhibitor of corneal lymphangiogenesis [94]. Besides MT1-MMP, the cornea also expresses MMP-2 and MMP-9. Using the selective inhibitor SB-3CT for MMP-2 and MMP-9, it has been demonstrated that also MMP-2 and MMP-9 are critically involved in corneal lymphangiogenesis during inflammatory response [95].

Aqueous humor is a clear body fluid in the anterior and posterior chamber of the eye. Its function is to supply the lens and the cornea with nutrients and remove potentially harmful agents. Moreover, it also contains several immunomodulatory factors. Just recently, we have shown that the aqueous humor exerts anti-hem- and anti-lymphangiogenic effects in vivo and in vitro [96]. Thereby, we have demonstrated that the immunomodulatory factors α-melanocyte-stimulating hormone (α-MSH) and vasoactive intestinal peptide (VIP) contained in the aqueous humor partially mediate the anti-lymphangiogenic effect [96]. These results demonstrated that aqueous humor contributes to the corneal (lymph)angiogenic privilege.

### 4.2. Non-Coding RNAs in Lymphangiogenesis

In recent years, non-coding RNAs (ncRNA) have gained more and more attention. NcRNAs are functional RNA molecules that have the ability to control gene expression. NcRNAs are divided into small/short ncRNAs (miRNA, piRNA, siRNA, etc.) and long ncRNAs (lncRNAs) [97]. Over the last few years, various miRNAs and lncRNAs that have an influence on hem- and lymphangiogenesis have been identified.

MicroRNAs (miRNA), a class of small ncRNAs of approximately 21–24 nucleotides (nt) in length [98,99], have been shown to play different roles in human organ transplantations [100,101]. However, there are only a few reports on miRNAs being directly associated with corneal transplant rejection.

The search for differentially expressed miRNA in isograft corneas vs. normal corneas, as well as in allograft corneas vs. isograft corneas could recently identify altered levels of four miRNAs in both groups [102]: miR-155-5p, miR-142-3p, miR-142-5p, and miR-223-3p. Using a penetrating keratoplasty model demonstrated high expression of miR-122 in the corneal stroma and it has been observed that the increased miR-122 expression significantly decreases the risk of corneal transplantation rejection [103]. However, further investigations are needed to understand the underlying mechanism.

In addition to their involvement in the rejection response of corneal grafts, miRNAs expressed in the cornea have recently been further identified to have a direct or indirect influence on lymphangiogenesis. Recently, a significantly reduced expression of miR-184 was observed in inflamed cornea compared to naive cornea. This is accompanied by reduced lymphangiogenesis [104]. Furthermore, overexpression of miR-184 in LECs in vitro suppresses adhesion as well as migration capacity and reduces the ability to organize into capillary-like tubes [104]. This also reveals that miR-184 is a negative regulator of the lymphangiogenic process.

Prox1, a transcription factor whose activity is important for lymphatic vessel development and maintenance [105,106,107], is mainly expressed in lymphatic endothelial cells and it has no influence on the development and function of blood vessels [107]. However, very little is known about the underlying mechanisms. When comparing embryonic blood endothelial cells with lymphoid endothelial cells, a significantly higher expression of miR-181a was observed. Furthermore, a specific consensus binding site for miR-181a was observed in the 3′ untranslated region (UTR) of Prox1. Direct binding of miR-181a to the 3′UTR of Prox1 results in rapid degradation of Prox1 mRNA. [108]. This way, miR-181 activity silenced Prox1 expression in the blood vasculature during development [108]. By determining the miRNA expression profiles of human LEC and blood endothelial cells (BECs), it could be shown that another miRNA (miR-31) is highly expressed in blood endothelial cells and binds to 3′UTR of Prox1 resulting in the degradation of Prox1 mRNA [109]. Just recently, miR-466 has been identified as a new prospect to target the 3′UTR of Prox1 [110].

VEGFR-3 proved to be important for developmental angiogenesis and lymphangiogenesis [111]. VEGFR-3 expression is maintained on all endothelial cells during development, but it becomes restricted to lymphatic endothelial cells in adulthood [112]. The transcription factor Prox1 [113] is decisive for VEGFR-3 expression, which explains the indirect regulation of VEGFR-3 in naive BECs by the above-mentioned miRNAs which regulate Prox-1. The mechanism, however, by which VEGFR-3 is controlled during inflammatory lymphangiogenesis is not completely understood. The induction of miR-1236 in lymphatic endothelial cells by the inflammatory cytokine IL-1β leads to translational inhibition of VEGFR-3, whereas the expression of VEGFR-2 is not altered [114]. On the other side, it was observed that miR-9 increases VEGFR-3 expression in LECs and promotes lymphatic tube formation [115]. More recently, miR-126 has been demonstrated to specifically regulates lymphatic development in part by modulating VEGFR-3 signal transduction. Also, LECs reacts poorly to VEGF-A and VEGF-C through reducing the expression of VEGFR-2 and VEGFR-3 after miR-126 inhibition. [116].

The Discoidin domain receptors family member 1 (DDRs)1 is expressed on epithelial cells, endothelial cells, and tumor cells [117,118,119] and interact with almost all types of collagen, including fibrillar collagens I–III [120,121]. Activation of DDR1 triggered by collagen-binding induces cell proliferation, tumor angiogenesis, and lymphangiogenesis [118,122]. Reduced expression of both the mRNA and the protein levels of DDR1 by miR-199a/b-5p lead to reduced tube formation in human LEC cells whereas an inhibitor of miR-199a/b-5p increases DDR1 expression and enhances tube formation in LECs. Moreover, the use of a corneal alkali-burn model, revealed that one subconjunctival injection of miR-199a/b-5p mimics not only suppressed DDR1 expression but also significantly reduced lymphangiogenesis in comparison to scramble control [118].

LncRNAs, on the other hand, are defined as transcripts longer than 200 nt that are not translated into protein. Several studies suggest that lncRNAs play a role in either negative or positive regulation of gene expression at both transcriptional and post-transcriptional levels in development and differentiation [123,124,125]. Many lncRNAs have been functionally associated with human diseases, particularly the development and progression of human cancers [126]. However, little is known about the expression of lncRNAs in the eye. Recently, it has been demonstrated that the expression pattern of lncRNA exhibits distinct changes during maturation between P0 and 8 weeks of age as well as tissue specificity within the cornea, lens, retina, RPE, choroid, and sclera. This result seems to be consistent with the idea that lncRNA may be important in maintaining tissue identity [127]. The involvement of lncRNA H19 in neovascularization has recently described with lncRNA H19 acting as a molecular sponge for miR-29c to regulate the expression of VEGF-A [128].

However, only a few studies to date are known that describe the influence of lncRNA in lymphangiogenesis. It has been shown that the antisense non-coding RNA in the INK4 locus (ANRIL) is significantly overexpressed in patients with colorectal cancer and correlates with higher expression of VEGF-C, VEGFR-3, and LYVE-1 [129]. The overexpression of lncRNA BLACAT2 (bladder cancer-associated transcript 2) promotes lymphatic metastasis by regulation of VEGF-C expression [130].

The data show that both miRNA and lncRNA are involved in the regulation of both hemangiogenesis and lymphangiogenesis. Therefore, they may be a promising therapeutic target. However, further studies are needed to better understand both miRNAs and lncRNAs in the regulation of hemangiogenesis and lymphangiogenesis.

## 5. Murine Cornea is a Suitable Model for Identification of Novel Endogenous Modulators of Lymphangiogenesis

A recent approach for the identification of new modulators of hem- and lymphangiogenesis is the comparison of naive or inflamed corneas of divergent mouse strains. With such an approach, the genetic heterogeneity of angiogenesis has been confirmed several years ago. The study found an up to a 10-fold difference in corneal blood vessel growth in response to FGF-2 and VEGF [131]. A follow-up study further analyzed the angiogenic responsiveness to FGF-2 and VEGF which led to the identification of several quantitative trait loci (QTL). QTL analysis is used to combine phenotypic information (e.g., vascular growth) and genotype data to identify chromosomal regions that contribute to the observed variation in the feature being analyzed. Thereby, they could identify six regions on chromosome 2, 4, 10, 13, 15, and 18 responsible for the VEGF and FGF-2 responsiveness [132,133].

**Figure 3:**
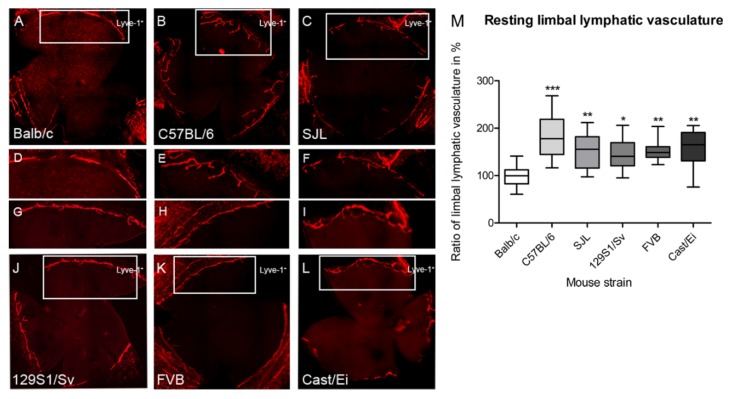
Lymphvascularized area of naive corneas from different inbred and wild-type mice strains. (**A**–**L**): Whole mounts of murine naive corneas stained with LYVE-1) with resting limbal vasculature (original magnification × 100). (A) BALB/c (B) C57BL/6N (C) SJL/J (J) 129S1/S (K) FVB/N (L) Cast/E. D–I) Higher magnification of the limbal vasculature of the strains. (**M**) Quantification of the area of the resting limbal lymphatic vasculature. Statistical analysis was done by Kruskal–Wallis test with Dunn’s multiple comparison post-test. * *p* < 0.05, ** *p* < 0.01, *** *p* < 0.001. (modified from [134]).

Since the discovery of lymphatic markers like LYVE-1 and VEGFR-3, research in genetic heterogeneity of lymphangiogenesis experiences more attention. Recently, we were able to identify further novel regulators of lymphangiogenesis by analyzing the differences between different mouse strains concerning lymphatic vessels could be found in a physiological situation. For that purpose, we determine the limbal lymphatic vascular and counted the number of the vascular extensions (sprouts) from the main limbal lymphatic vessel into the avascular cornea from whole mounts of naive eyes. The determination of the total area of the limbal lymphatic vessel and the physiological extension showed that BALB/c has the lowest resting limbal lymph vascular area. The area of the resting limbal vasculature varied significantly between 1.4-fold for 129S1 and 1.8-fold for C57BL/6 compared to BALB/c (Figure 3). We also observed significant differences in the number of physiological extensions in the avascular cornea. The numbers of extension into the avascular cornea are 1.5-fold higher in C57BL/6 and 1.6-fold higher in SJL and FVB in comparison to BALB/c [134]. Furthermore, we compared the limbal lymphatic vasculature in corneal inflammatory lymphangiogenesis and VEGF-C induced lymphangiogenesis to analyze the presence of strain-dependent differences in lymphatic vessel growth. The results clearly show that, depending on the mouse strain, the lymph vascular surface induced by VEGF-C varied up to 1.9-fold. The lymph vascular surface induced by the suture-induced inflammatory corneal neovascularization model also varied up to 1.7-fold depending on the mouse strain. This shows that the genetic background is an important predictor for the extent of growth factor-induced (VEGF-C) and inflammation-induced lymphangiogenesis. The data shows significant differences in the lymphangiogenic response between the different classical and wild-derived inbred strains and this suggests underlying genetic factors influencing the lymphangiogenic response [134].

To analyze whether the phenotypic differences correlate in the differential expression of individual genes between the strains, we compared the naive cornea of the highly lymphangiogenic C57BL/6 mouse and the low lymphangiogenic BALB/c mouse with the pathway-specific expression analysis. We identified 13 differentially regulated genes in the cornea of C57BL/6 mice compared to BALB/c mice. Among the identified genes were already known genes that affect the lymphangiogenesis, such as thrombospondin. This shows that the method is suitable for identifying new endogenous modulators. Two of these thirteen differently expressed genes, *tumor necrosis factor (ligand) superfamily member 10 (Tnfsf10/Trail)* and *plasminogen activator tissue (Plat/tPA)*, showed markedly higher expression in the BALB/c mice compared to the C57BL/6 animals. Furthermore, we identified that besides mRNA expression Trail and tPA are also expressed on protein level in the cornea of both strains. Moreover, we could demonstrate that both Trail and tPA inhibit the proliferation of LECs [135]. The data demonstrate that Trail and tPA may contribute to the lymphangiogenic privilege of the cornea.

**Figure 4:**
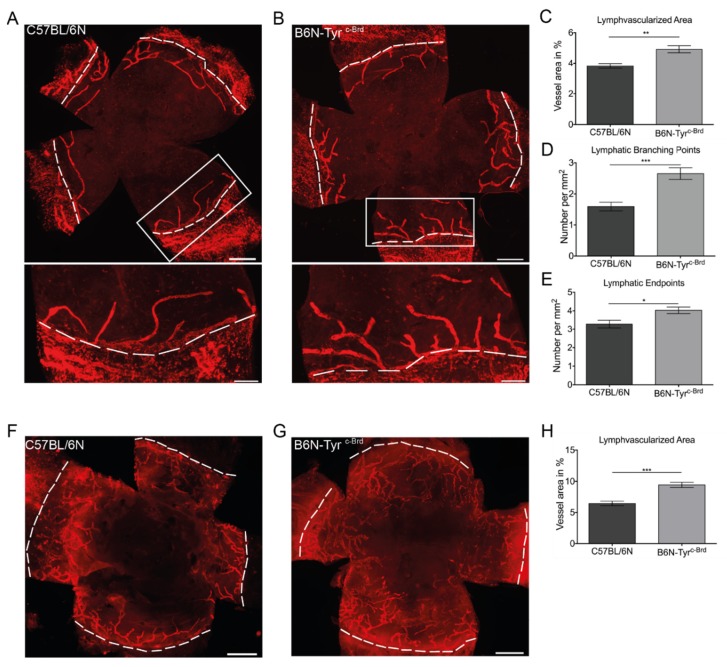
Absence of tyrosinase leads to increased developmental and inflammation-induced lymphangiogenesis (comparing C57BL/6N with B6N-Tyr^c-Brd^). (**A**) and (**B**) Corneal whole mounts of the murine cornea from C57BL/6N (**A**) and B6N-Tyr^c-Brd^ (**B**) stained for LYVE-1. The boxed areas show in higher magnification of the limbal vasculature in the bottom panels. (**C**) Quantification of the lymphatic vascularized area of the whole mounts and determination of the number of branching points (**D**) and the number of endpoints (**E**). Results are presented as the average of C57BL/6N and B6N-Tyr^c-Brd^ mice. F) and G): Corneal whole mounts of inflammation-induced lymphangiogenesis of C57BL/6N (**F**) and B6N-Tyr^c-Brd^ (**G**) stained for LYVE-1. (**H**) Quantification of the inflammatory lymphatic vascularized area of the whole mounts. Dashed lines show the border between the limbus and the cornea. Statistical significance was calculated by two-tailed t-test. Data are expressed as means SEM. A, C-E *n* = 7 C57BL/6N mice; B, C-E n = 6 B6N-Tyr^c-Brd^ mice; F-H *n* = 6. * *p* < 0.05, ** *p* < 0.01, and *** *p* < 0.001. Scale bars: 500 mm (**A** and **B**, upper panels, **F**, and **G**); 200 mm (**A** and **B**, lower panels). Original magnification, 100× (**A**, **B**, **F**, and **G**). (adopted form [137]).

Lymphangiogenesis is a quantitative trait that can be reliably quantified by the determination of morphometric parameters in the mouse cornea [136]. Using this, two phenotypically different mouse strains, the low-lymphangiogenic BALB/cN and the high-lymphangiogenic C57BL/6N, were crossed to generate a population showing genetical and phenotypic variance. To perform a QTL analysis, five morphometric parameters, the lymph vessel area, the lymph vessel length, the number of vascular extensions from the major limbal lymph node, the number of endpoints, and the number of branching points, each relative to the total corneal area of interest, were determined in a large BALB/cN x C57BL/6N F2 intercross (*n* = 795), were determined and related to the cornea. Based on the results of the QTL analysis a particular strong genome-wide significant locus was identified on chromosome 7 containing *tyrosinase* as a potential new candidate gene contributing to the differences in limbal lymphatic vessel architecture in BALB/c and C57BL/6 mice. To validate that tyrosinase is involved in lymphangiogenesis C57BL/6 mice were compared with C57BL/6 mice carrying an albino mutation in the *tyrosinase* gene. Albino C57BL/6 (B6N-Tyr^cBrd^) mice showed significantly increased limbal lymph vascularized areas, a higher number of lymphatic vessel endpoints and branching points in the naive cornea compared to C57BL/6 mice. In addition, the albino C57BL/6 mice also showed increased inflammation-induced lymphangiogenesis in comparison to C57BL/6 animals. These findings confirm that tyrosinase is a novel regulator of lymphangiogenesis in developmental and inflammatory lymphangiogenesis [137] (Figure 4).

These results clearly demonstrate that genetically distinct mouse strains are useful for identifying new moderators of lymphangiogenesis, like Trail, tPA, and tyrosinase, and that the cornea is an ideal model for identifying these modulators.

## 6. Conclusions

In recent years, it has been clearly demonstrated that, in addition to blood vessels, the non-visible lymphatics are also involved in the rejection reaction of corneal transplants. The modulation of the blood and lymph vessels before and after cornea transplantation, therefore, represents a good therapy option to improve the survival of the graft. In addition to the already known modulators, further new regulators of lymphangiogenesis (Table 1) have been described in recent years using increasingly sophisticated analytical methods and procedures. In addition to various proteins, these include ncRNA (miRNA, lncRNA) which also influence lymphangiogenesis. These novel identified factors may be promising therapeutic targets for the treatment of pathological lymphangiogenesis in a variety of ocular and extraocular diseases such as graft rejection, tumor metastasis, and dry eye disorders.

## Figures and Tables

**Table 1:** Proteins and non-coding RNA (ncRNA) involved in regulating (lymph-)angiogenesis.

Proteins in Lymphangiogenesis			
	Protein	Function	
Endostatin	Endostatin	inhibition of angiogenesis	[32,37,38,39]
	Tumstatin	inhibition of angiogenesis	[40,41]
	Arrestin	inhibition of angiogenesis	[41]
Plasminogen	Angiostatin	inhibition angiogenesis	[42,43]
Thrombospondin	TSP-1	inhibition of angiogenesis and lymphangiogenesis	[44,83]
	TSP-2	inhibition of angiogenesis	[31]
soluble VEGFR	sVEGFR-1	decoy receptor for VEGF-A; inhibition of angiogenesis	[28,46]
	sVEGFR-2	decoy receptor for VEGF-C and -D; inhibition of lymphangiogenesis	[47]
	sVEGFR-3	decoy receptor for VEGF-C and -D; inhibition of lymphangiogenesis	[48]
adapter protein	IRS-1	treatment with antisense oligonucleotide inhibits hem- and lymphangiogenesis	[65]
Glycoprotein	Podoplanin	implication in lymphocyte trafficking, blocking antibody inhibits lymphangiogenesis	[71,72]
Integrine	Integrin α_5_β_1_	treatment with antagonist JSM6227 inhibits lymphangiogenesis	[3]
	Integrin α_9_β_1_	blocking antibody improves graft survival	[75]
Semaphorine	Semaphorin-3F	contributing to anti-(lymph) angiogenic barrier	[82]
Vasohibin	VASH-1	negative feedback; regulator inhibition of angiogenesis and lymphangiogenesis	[84,86]
transmembrane Receptor	Neuropilin-2	associated with VEGFR-3, artificial miRNA improves graft	[87]
Metalloproteases	MT-MMP1	cleavage of VEGFR-1 and LYVE-1	[91,94]
	MMP-2 & MMP9	blockade with SB-3CT inhibits lymphangiogenesis	[95]
Peptide hormone	VIP	inhibition of lymphangiogenesis	[96]
	α-MSH	inhibition of lymphangiogenesis	[96]
TNF/TNFR-Superfamily	Trail	inhibition of lymphangiogenesis	[135]
Proteases	tPA	inhibition of lymphangiogenesis	[135]
Membrane protein	Tyrosinase	inhibition of lymphangiogenesis	[137]
**ncRNAs in Lymphangiogenesis**			
	Targets	Function	
miRNA-184	LECs	suppresses migration and adhesion	[104]
miRNA-181a	Prox-1	degradation of Prox-1	[108]
miRNA-31	Prox-1	degradation of Prox-1	[109]
miRNA-466	Prox-1	degradation of Prox-1	[110]
miRNA-1236	VEGFR-3	inhibition of VEGFR-3	[114]
miRNA-9	VEGFR-3	increased VEGFR-3 expression	[115]
miRNA-126	VEGFR-2/VEGFR-3	modulates VEGFR-2 and VEGFR-3 signal transduction	[116]
miRNA-199a/b5p	DDR1	degradation of DDR1	[118]
